# Imperfect Implementation of an Early Warning Scoring System in a Danish Teaching Hospital: A Cross-Sectional Study

**DOI:** 10.1371/journal.pone.0070068

**Published:** 2013-07-26

**Authors:** Mark Niegsch, Maria Louise Fabritius, Jacob Anhøj

**Affiliations:** 1 Anaesthesiology Department Z, Bispebjerg Hospital, Copenhagen, Denmark; 2 Centre of Head and Orthopaedics, Anaesthesiology Department 4231, Copenhagen University Hospital, Copenhagen, Denmark; 3 Danish Society for Patient Safety, P610 Hvidovre Hospital, Hvidovre, Denmark; D'or Institute of Research and Education, Brazil

## Abstract

**Background:**

In 2007, the initiation of a patient safety campaign led to the introduction of Ward Observational Charts (WOC) and Medical Early Warning Score (MEWS) at Naestved Regional Hospital. This included systematic measuring of vital signs of all patients in order to prevent patient deterioration and assure timely and correct initiation of treatment. The aim of this study was to assess to what degree WOC guidelines being followed by ward staff.

**Design and Setting:**

A 7-day prospective, observational, randomised, cross-sectional, point prevalence study of WOC guideline compliance in hospitalised patients on twelve wards at Naestved Hospital.

**Results:**

The study included 132 patients. Of these, 58% had been observed and managed correctly according to WOC guidelines. 77% had all MEWS elements recorded by staff. One patient had no MEWS elements recorded. Only 38% of patients with abnormal MEWS were correctly escalated by nursing staff. Staff was aware of the abnormal MEWS observed by investigator in 60% of the patients. Each element of WOC was on average recorded by staff in 90% of the patients.

**Conclusion:**

At the time of our study, the long-term implementation of WOC guidelines has not been completed satisfactorily. The lacking component in the implementation of MEWS and WOC is the documentation of action taken upon finding an abnormal value. Unsuccessful implementation could result in incorrect results from evaluation of an early warning system. We suggest a redesign of the training programme to educate staff in recognising and caring for critically ill patients at Naestved Hospital.

## Introduction

Adverse events such as cardiac arrest, unexpected intensive care unit (ICU) admittance and unexpected death among hospitalised patients are often preceded by abnormal physiology[1]–[8]. This is manifested as alterations in vital signs [9]. Fuhrman et al. have found that almost half of the patients with abnormal vital signs are unrecognised by the ward staff [3], [10].

Inspired by organisational changes in Australia, the United States of America and the United Kingdom, medical emergency teams (MET) and medical early warning signs (MEWS) were introduced at Naestved Hospital as a part of a national patient safety campaign called Operation Life.

Reports on the consequences of MET have been conflicting [11]–[15]. However, recent studies have found positive effects on reducing adverse effects, [16], [17] unexpected ICU admission and mortality [18]. Mitchell et al. have found an increased number of reviewed patients with significant clinical instability, increased numbers of MET reviews and better documentation of vital signs [18].

Naestved Regional Hospital is a 362 bed, acute care hospital with teaching function. MET and ward observation chart (WOC) were introduced between 2007 and 2009. The aim was to prevent unrecognised deterioration and adverse effects among hospitalised patients. Nurses and nursing students made the departmental observations. Nurses contacted the ward physicians when dictated by WOC guidelines.

WOC is an observation chart on which MEWS is calculated and furthermore it includes the escalation protocol upon encountering deteriorating patients. Parameters included in the WOC are: respiratory rate (RR), blood pressure (BP), heart rate (HR), temperature (TP), Glasgow Coma Scale (GCS) and transcutaneous oxygen saturation (SpO2).

In 2010, WOC guidelines were adjusted so that all patients were to have their MEWS recorded at least 3 times daily during the first 24 hours of admission unless a doctor decided otherwise. If MEWS was 0 during the first 24 hours, MEWS recording frequency could be reduced to once a day. Mandatory education of all existing and new staff in recognition and treatment of critically ill patients was introduced as a 2- hour lecture, including instructions in the use of WOC and MET.

**Figure 1:**
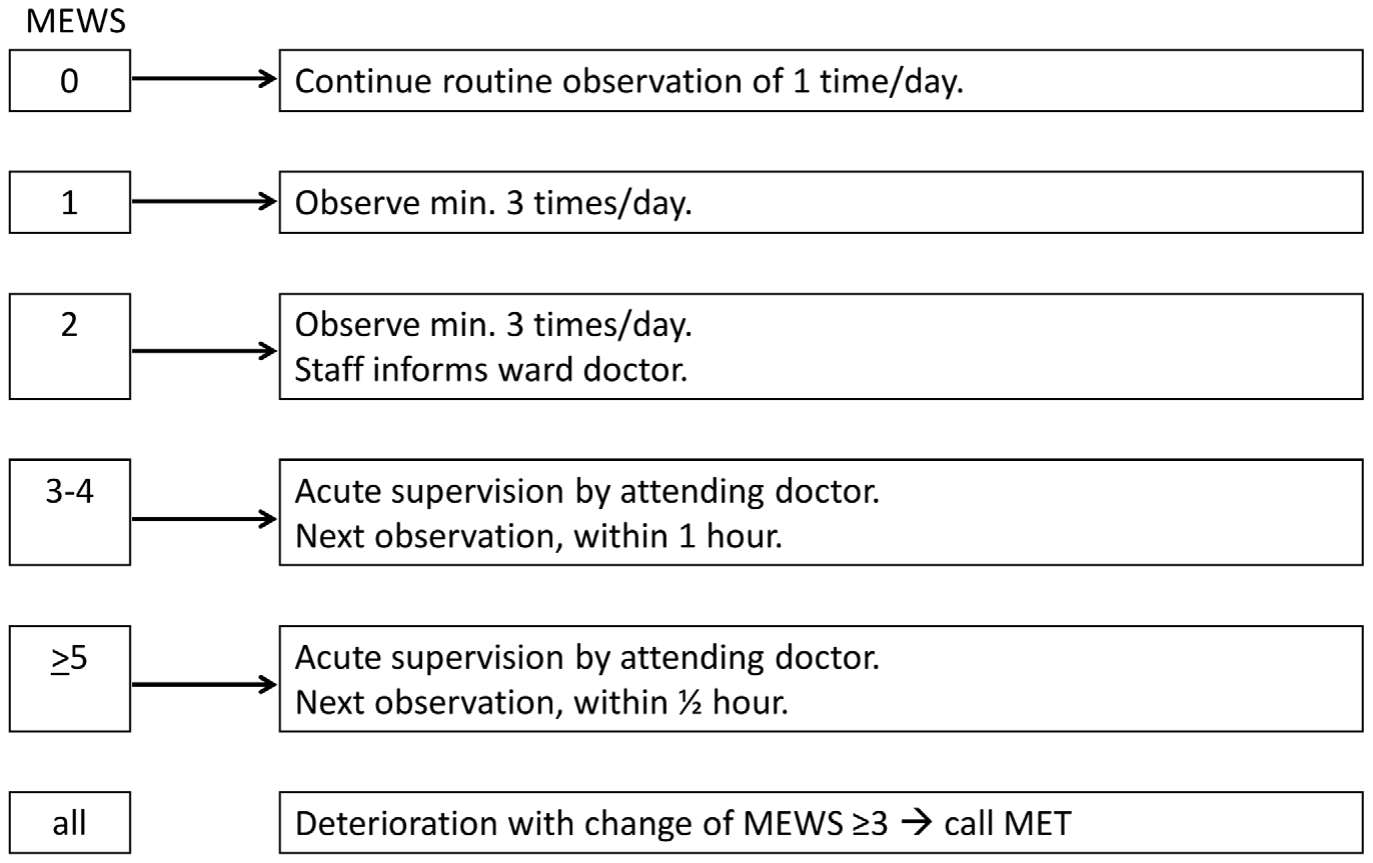
WOC escalation protocol. For each summarised MEWS value the appropriate actions to be taken are found at the right. Regardless of the prior MEWS recorded, a sudden change in MEWS of 3 or more triggers activation of MET.

The vital signs were recorded on the WOC where a graphical layout with color-coding facilitated easy reading and calculation of the Medical Early Warning Score (MEWS) (table 1). The more abnormal the parameters were, the higher a MEWS the patient received. The MEWS dictated which actions to be taken in accordance with the WOC escalation protocol (figure 1).

**Table 1:** Calculation of MEWS.

Vital sign	MEWS value = 0	MEWS value = 1	MEWS value = 2	MEWS value = 3
RR	10–20	9 or 21–30		≤8 or >30
^*^SpO_2_	≥93%	90–92%		<90%
TP (°Celsius)	≥36-≤38°	35–36° or >38–39°	<35° or >39°	
BP, mmHg (systolic)	100–199	90–99	≥200	<90
HR	50–90	40–49 or 90–110	110–130	<40 or >130
GCS				Reduction ≥2

Assignment of MEWS single parameter values according to the recorded vital signs. MEWS is calculated by summation of all single parameter values. *For patients with pulmonary disease, expected SpO2 can be reduced by 5%.

Furthermore, the call criteria for the MET allowed activation upon concern for a patient, even if there were no abnormal vital signs. The aim of this study was to assess to what degree WOC guidelines were being followed by ward staff.

## Materials and Methods

### Study Design

We conducted a 7-day (Monday-Sunday) observational, randomised, cross-sectional, point prevalence study of hospitalised patients on 12 wards at Naestved Hospital. Each hospital bed on the included wards was randomised using a random number generator to a specific weekday [19]. The wards included were surgery, gynaecology, orthopaedics, cardiology, pulmonology, endocrinology, oncology, neurology, geriatrics, and acute medicine. A total of 269 beds were included.

Each day of the study between 16∶00 and 21∶00 all WOC parameters (BP, HR, SpO2, RR, TP and GCS) were recorded and MEWS calculated by investigator. This was done to all patients present in the beds randomised for that day. All empty beds were excluded. Reasons for the empty beds were: patients at examinations and surgery, patients on leave during the time of data collection, patients who did not want to participate, patients under strict isolation, terminal patients and deceased patients.

Data collection was performed by nursing students (investigators) who had received training in proper use of WOC and measurement of vital signs. Training included a 2- hour training session on data collection and structured interviews of ward nurses. Data were collected on paper forms for later entry into a designated database.

Investigator used a structured questionnaire to interview the ward nurse whenever a patient had abnormal MEWS in order to asses if the ward nurse was aware of the patient’s condition.

Each patient’s WOC was copied in order to compare investigator MEWS to staff MEWS and in order to determine the level and frequency of MEWS for the previous 48 hours.

### Ethics

The study was a non-intervention study, but staff was informed of any abnormal vital signs found by investigator and all patient data were anonymised.

The regional Danish Data Protection Agency approved the study. Since it was an observational, non-intervention study and was not under the law of a research ethics committee system, patients were only required to give verbal informed consent. Six patients did not give their verbal consent and investigators were instructed to discontinue the data collection and the beds were excluded from the study.

### Data Collection and Management

The primary outcome of the study was:

1 The proportion of all in-hospital patients who were observed and managed in accordance with WOC guidelines.

Secondary outcomes were:

2 The proportion of all patients who had each MEWS element recorded by staff,
3 The proportion of patients with MEWS calculated by staff,
4 The proportion of patients with abnormal MEWS (>0) recorded by staff with documentation of appropriate action taken in accordance with WOC guidelines,
5 The proportion of patients with abnormal MEWS recorded by investigator with staff aware that the patient had abnormal MEWS,
6 Interobserver agreement (Cohen’s Kappa) between staff and investigator regarding abnormal MEWS.

For each patient, the following variables were recorded and entered into a Microsoft Excel 2003 spread sheet:

1. Patient ID
2. Weekday of data collection (Monday – Sunday)
3. Department
4. Latest MEWS value within 48 hours recorded by staff (0–6/missing)
5. MEWS recorded by investigator (0–6)
6. Abnormal MEWS recorded by investigator known to staff (yes/no/not relevant)
7. Abnormal MEWS recorded by staff with documented appropriate action (yes/no/not relevant)
8. MEWS recorded by staff and patient managed in accordance with WOC guidelines (yes/no)
9. For each MEWS element, element recorded by staff (yes/no)

Due to lost documentation, data elements 6, 7, and 8 were missing for 5, 6, and 7 patients, respectively. These patients were included in the intention-to-treat population, i.e. they were included in the denominators but not the nominators of the relevant outcome measures.

Two patients were excluded from the data analysis because no data were recorded on the investigator data collection form.

In order to comply with WOC guidelines, all single elements of the MEWS had to be recorded, a total MEWS calculated and registered, followed by correct adjustment of observation frequency and documented contact to physician or MET, if dictated by WOC guidelines. All was tobe documented.

### Statistical Analysis

Since all in-hospital patients were included in this study, the outcome measures 1 to 5 were reported as crude numbers and percentages and the calculation of confidence intervals were deemed neither useful nor appropriate.

Data were analysed and graphs were constructed using R Statistical Software v. 2.13.1.

The overall WOC compliance (outcome 1) was further stratified and compared by department and day of week using p-charts. A p-chart was also used to compare the compliance with single elements of WOC (measure 9).

A p-chart is a type of control chart. Control charts are generally used to describe the magnitude and type of variation over time or, as in this case, between subgroups (departments, weekdays and WOC elements). Variation can be of two types: Common cause variation (random variation or noise) is part of any process. Special cause variation (non-random variation or signal) is present if some subgroups deviate significantly from the average performance of all subgroups. On the control chart special causes are identified by data points outside the control limits of the chart. By convention, control limits are set at average+/−3 x the estimated within subgroup standard deviations [20].

## Results

**Figure 2:**
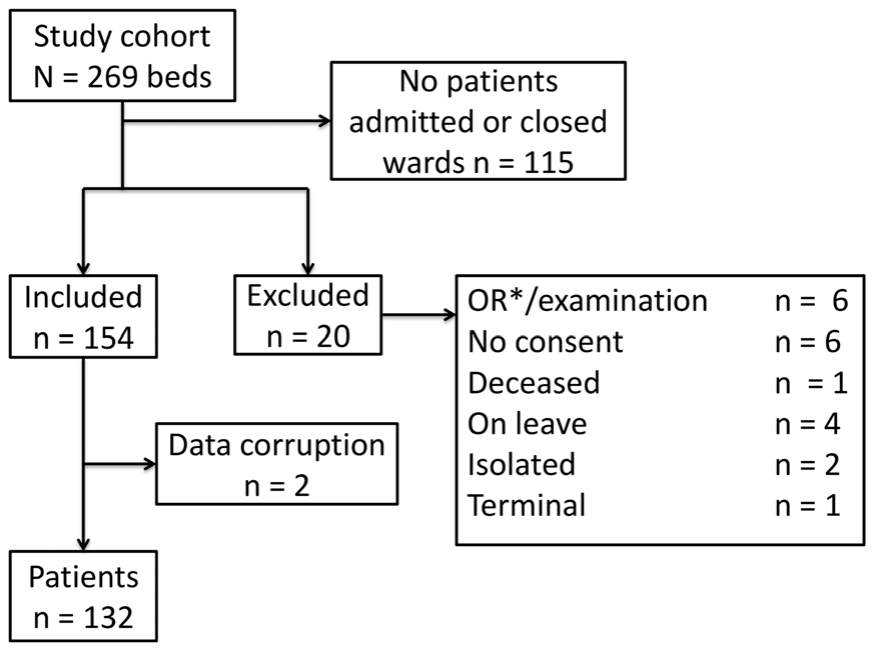
Distribution of study cohort. Distribution of study cohort (n = number of patients). *OR (operating room).

**Figure 3:**
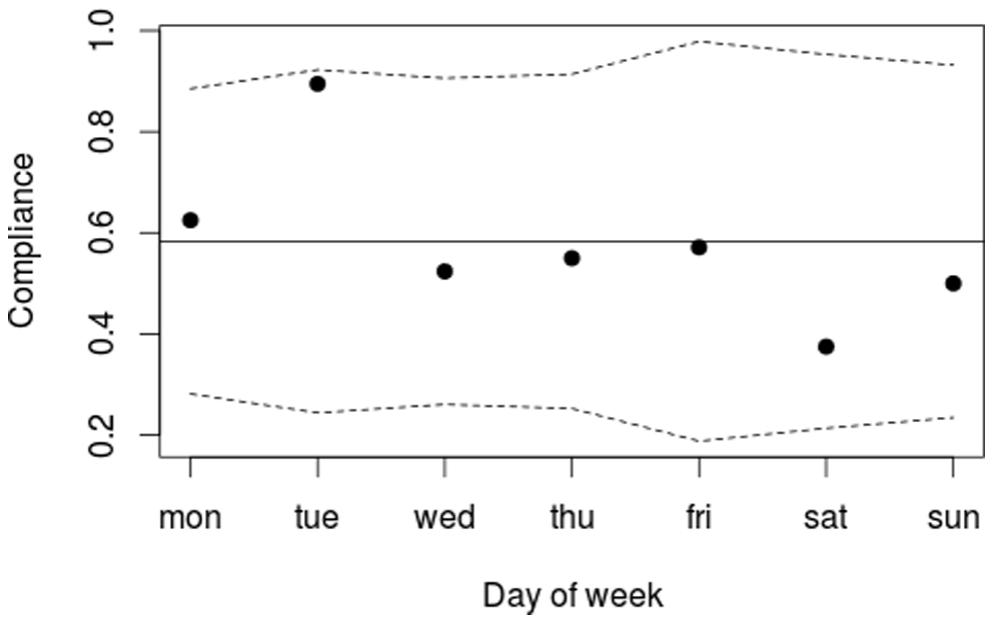
WOC compliance by weekday. The proportion of patients who were observed and managed according to WOC guidelines. Each data point shows WOC compliance measured on a day of the week. Compliance is defined as the proportion of patients who had all elements of the measure completed. The dashed lines are control limits showing the range of common cause variation. The figure shows only common cause variation, i.e. there is no sign of compliance on any day of the week deviating significantly from the average.

The study included 132 patients (figure 2). Of these, 77 (58%) had been observed and managed correct according to WOC guidelines. There was no evidence of significant difference in this measure between departments or day of week (figure 3).

One hundred and one (77%) patients had MEWS calculated by staff at least once within 24 hours prior to investigator’s visit. Curiously, 12 of the patients with calculated MEWS did not have all vital parameters necessary for calculation of the MEWS recorded. Twelve patients without MEWS had all necessary parameters recorded.

One hundred and one patients (77%) had all MEWS elements recorded by staff. One patient had no MEWS elements recorded.

A total of 50 patients had documented abnormal MEWS recorded by staff. Of these, 19 (38%) had documented appropriate action taken according to WOC guidelines.

Seventy-three patients had abnormal MEWS recorded by investigator and in 44 (60%) of these patients, staff was aware that the patient had abnormal MEWS.

**Figure 4:**
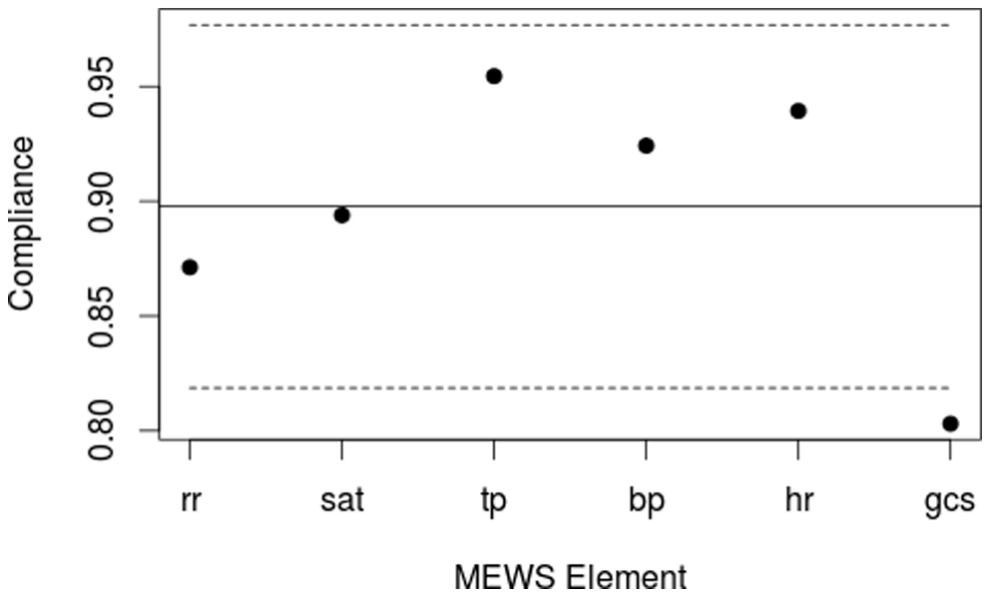
MEWS compliance by MEWS element. Compliance defined as the proportion of patients who had MEWS single elements measured at least once during previous 24 hours according to WOC guidelines. rr = Respiratory Rate, sat = Oxygen Saturation, tp = Temperature, bp = Blood Pressure, hr = Heart Rate, gcs = Glasgow Coma Scale. The dashed lines are control limits showing the range of common cause variation. One data point, GCS, is outside the control limits indicating that compliance with GCS measurement is significantly lower than with other elements.

Each single element of the WOC had on average been recorded by the staff in 90% of all patients. The elements were recorded as follows: RR 115/132(87%), SAT 118/132(89%), TP 126/132(95%), BP 122/132(92%), HR 124/132(94%), GCS 106/132(80%). Recording of GCS was significantly lower than for other elements (figure 4).

Among the structured interviews with nurses concerning the 31 patients with abnormal MEWS and no action documented, 27(87%) gave an answer to why no action had been documented after encountering abnormal MEWS which would normally require contact to ward physician or MET. Among the standardised answers, 9 answered that the abnormal vital signs were normal for the specific patient, 5 answered that the patient was known to have abnormal vital signs and 2 answered that the patient appeared well-being despite the abnormal vital signs. 4 patients were observed after initiation of treatment, 2 answered that they were observing the patient closely and in one case the nurse answered that he/she would check up on the patient later. Individual answers included 1 later assessment of the patient due to recent activity and omission of MEWS because another observation chart was in use (2 patients), because the patient was terminal (1 patient) or because the WOC had been discontinued (1 patient) (table 2).

**Table 2:** Results of the standardised interview.

These abnormal vital signs are normal for this patient	9
This patient is known with abnormal vital signs	5
This patient appears well-being despite the abnormal vital signs	2
This patient has started treatment and awaits effect	4
I am watching this patient and his vital signs	2
I will check up on the patient later	1
*Individual answers:*	
Staff assesses the patient later, due to recent physical activity	1
The patient is assessed with a different observation chart	2
The patient is terminal	1
WOC has been discontinued	1

Answers from the awareness interview with staff who had not documented a reaction to the abnormal MEWS. The first 6 answers were pre-printed on the interview questionnaire.

Supporting material is available as web material in the form of raw Data S1, along with an explanation of the Data S2.

## Discussion

We found that progress has been made in the systematic observation of hospitalised patients and most patients had their vital parameters measured at least once a day. However, overall documentation of compliance to WOC guidelines including documentation of acting on abnormal MEWS was low at the time of our study. Structured interviews indicated that there had been reflection in several cases where no action had been taken upon encountering abnormal MEWS.

In order for the guidelines to work, a very high compliance at all steps of the system is crucial (figure 1). At the time of our study, we found that almost all patients had one or more vital signs recorded in accordance with the guidelines and that registration of single elements of the MEWS was on average 90%. However, one or more steps of the WOC escalation protocol were undocumented in 42% of patients. Only 77% had all MEWS parameters recorded, and 77% had MEWS calculated. Interestingly, some patients had their MEWS calculated even if they missed one or more elements necessary for the calculation. Finally and most importantly, only 38% of patients with abnormal MEWS had documented appropriate action taken according to WOC guidelines.

Fuhrmann et al. have observed that among patients with abnormal vital parameters collected by investigator only between 66% –77% had one or more vital parameters recorded by staff [10]. Fuhrmann et al. have found that none of the patients with abnormal vital parameters had their RR measured [10]. RR is considered to be of special importance [21]. RR is not an overlooked element at Naestved Hospital, but an element, which is recorded equally to other vital parameters.

Compared to the results found by Fuhrmann et al., we found that a higher proportion of patients had their vital parameters recorded. However, at the time of our study the documentation of overall management of patients with early signs of deterioration was still not in accordance with WOC guidelines in 62% of the patients. This suggests that the main problem does not lie within the recording of vital parameters, but somewhere in the process of reflecting, acting or documenting when encountering abnormal vital parameters. This questions the value of our present strategy including how we train existing and new staff and in the use of WOC guidelines.

Since this study is only designed to determine the compliance with WOC guidelines rather than to explain why the guidelines are not being followed, we can only speculate on the reasons for our findings. For this, we find it helpful to discriminate between the reasons why staff does not record vital signs and summarising them with MEWS and the reasons why staff does not document actions taken in accordance with WOC guidelines. In this study, recording of vital signs were considerably higher than in previous studies. Still, at the time of our study, recording of GCS was significantly lower. We believe, that the main reason for this is that ward staff in general is not accustomed to the use of GCS. As the use of GCS becomes standard this might change similar to what we have observed with RR. For most patients, the recording of GCS is straightforward and takes only a few seconds to complete.

Reasons for not summarising vital signs by calculation of MEWS may be that staff are not aware of the importance of MEWS [22] or that staff may believe that MEWS is only necessary if there are abnormal vital signs. Staff answers to the structured interview (table 2), indicated that they had been reflecting upon the elevated MEWS recorded, but it had not been documented anywhere. 87% gave a reason for not acting on abnormal MEWS. It is possible for a physician to deviate from the WOC escalation protocol but it requires an ordination in the patients record. If a patient is known to have abnormal vital signs for example due to chronic obstructive lung disease or atrial fibrillation, it is reasonable to adjust the acceptable intervals in the patient record. In the same way, a plan should be entered into the patient record whenever a physician decides to deviate from WOC after initiating treatment and awaiting an effect. Sixteen (60%) answers indicated that deviation from protocol was due to known or expected abnormalities in the patient’s vital signs. In 4 (15%) patients, answers showed that MEWS was terminated but this was just not entered into the patient records. These findings show that in 75% of the patients that were not observed correctly according to the WOC escalation protocol, the problem is a lack of ordinations from the physician regarding acceptable deviation from protocol or termination of observation. One patient had his measurement of vital signs postponed due to recent physical activity, which was a relevant observation and action taken by the nurse. This leaves 7 patients, 4 who were awaiting effect of treatment, and 3 patients who nurses stated that they were observing and following up on. For patients who have had a recent treatment initiated, it is important to follow the changes in vital signs in order to evaluate whether the initiated treatment has had the desired effect or if it is necessary to reconsider the treatment. Patients with septicaemia are dependent on quick and correct treatment, which has to be evaluated often in order to recognise under-treatment and therefore it is a necessity with close observation after initiation of treatment with fluids and antibiotics. The last 3 answers stated that nursing staff was observing the patient and if so it is merely a question of recording the measured vital signs.

pIn other studies, nurses have stated that they use vital signs only to support their own clinical judgement [23]. This may explain some of the difficulties with making nurses accept the routine measurement of vital signs and documentation of action. On the basis of the answers to the standardised interview, it seems that this is not the main problem at Naestved Hospital. In most cases there actually is a correct reflection and in some cases even a correct action but it is never documented. However, this is in itself a problem and a sign of poor quality of care.

Other reasons for not documenting or summarising vital signs have been cited as poor observational chart design [24]. Our WOC has been developed over several years in a continuous and iterative process involving ward staff at all levels. Therefore, we do not think that our WOC design is a barrier for documenting MEWS at Naestved Hospital.

We think the main reason for staff not reacting to abnormal MEWS or adjusting observation frequency when indicated is a combination of factors including lack of understanding the importance of abnormal MEWS and lack of knowledge of what actions to be taken whenever MEWS triggers an action. These considerations lead back to the training programme, which all clinical staff must complete.

At present, the programme is presented in lecture form. Mitchell et al. have found that extensive education including e-learning and participating in simulations of patient scenarios had a marked effect on staff compliance to the early warning system [18]. In order to facilitate better reflection and compliance to WOC guidelines, we suggest a redesign of the WOC training programme for all staff at Naestved Hospital including elements from e-learning and simulation.

Most evaluations of early warning systems have not included an assessment of the degree of implementation[11]–[17]. Other studies have mentioned the implementation but do not account for this when concluding upon the results [18]. The degree of implementation could have an impact on the results of such an evaluation. Most likely it would result in a false negative or reduced effect of introducing an early warning system. Therefore, it seems likely that any evaluation and comparison of early warning systems would require an initial assurance of a successful implementation after a period long enough to minimise the Hawthorne effect.

In recent years, electronic calculation of EWS and even measurement of vital signs have been introduced. Since our study finds a main problem in the recording and acting upon abnormal vital signs, it is reasonable to consider that an electronic calculation of EWS and perhaps automated alert would improve the outcome. Electronic calculation of EWS has been proven more precise and faster than the use of pen and paper [25], [26]. We found 12% of the EWS which had not been recorded or calculated correct and for these it could have made a difference if the calculation had been electronic and prompted staff for all vital signs before calculation. Also 2 patients in our study were not observed with WOC because a different observation chart was used. By using the electronic calculation of EWS it is possible to assure a national uniform way of collecting data and then extracting an EWS score, as seen in the test of the VitalPAC™ where the level of consciousness can be assessed both by the AVPU (alert, verbal, Pain and Unresponsive) and GCS score [27]. One step further and more expensive is the introduction of automated collection of vital signs, calculation of EWS and automated alerting of staff, which could reduce the length of hospital stays by improving EWS accuracy and clinical attendance to unstable medical patients [28].

In conclusion, progress has clearly been made in the observation of patients’ vital signs, however, we still believe that the implementation of WOC guidelines at Naestved Hospital has not been completed satisfactorily at the time of our study, mainly because of the lack of documentation of relevant actions taken and a lack of relevant ordinations regarding deviation from the WOC observation protocol. Further evaluation of this early warning system, should await complete implementation in order to avoid misleading results. Our study could have important implications for future clinicians in their process of implementation and evaluation of an early warning system.

We suggest a redesign of the training programme to educate staff in recognising and caring for critically ill patients at Naestved Hospital.

### Limitations

As a single cross-sectional design, this study did not allow for time series analysis of WOC compliance over time. During data collection, several wards were closing down due to a planned rehousing of beds between several regional hospitals. Also, data collection was done during a holiday week where some wards and beds were closed.

### Strengths

The study included all wards to which WOC guidelines were considered implemented. To eliminate any weekday effect, data collection was spread over a full week and all beds were randomised to a single day. This study was performed after an implementation period of several years, which means that any positive effect observed in the first period of implementation (Hawthorne effect) would have passed.

## Supporting Information

Data S1 **Raw Data.** This file contains all raw data used for this article.
(XLSX)

Data S2 **Data Explanation.** This file contains an explanation for understanding the raw data presented in, S1.
(TXT)
